# Oxidative Stress in Infection and Consequent Disease

**DOI:** 10.1155/2017/3496043

**Published:** 2017-02-01

**Authors:** Alexander V. Ivanov, Birke Bartosch, Maria G. Isaguliants

**Affiliations:** ^1^Engelhardt Institute of Molecular Biology, Russian Academy of Sciences, Vavilov Str. 32, Moscow 119991, Russia; ^2^Cancer Research Center Lyon, INSERM U1052 and CNRS 5286, Lyon University, 69003 Lyon, France; ^3^DevWeCan Laboratories of Excellence Network (Labex), Lyon, France; ^4^Riga Stradins University, Riga LV-1007, Latvia; ^5^Department of Microbiology, Tumor and Cell Biology, Karolinska Institutet, 17177 Stockholm, Sweden; ^6^NF Gamaleja Research Center of Epidemiology and Microbiology, Moscow 123098, Russia

Viral, bacterial, and parasitic infections comprise a vast group of etiological agents that cause acute or chronic diseases. According to WHO, they represent one of the major causes of human morbidity and mortality. AIDS, lower respiratory tract infections, and diarrheal diseases underlie up to 5 million deaths each year, especially in the middle- and low-income countries. Some of the infections causing chronic disease lead to the development of an array of deadly pathologies including cancer, autoimmune diseases, diabetes mellitus, and malfunctions of various organs. During the last two decades it has been clearly established that many of these infections trigger the production of reactive oxygen (ROS) and nitrogen (RNS) species. This is particularly true for infections caused by the blood-borne hepatitis viruses (B, C, and D), human immunodeficiency virus (HIV), influenza A, Epstein-Barr virus, respiratory syncytial virus, and other viruses. For acute respiratory viral infections, ROS/RNS have been implicated in lung tissue injury and epithelial barrier dysfunction which in turn increased the susceptibility to secondary infections. In case of chronic viral hepatitis, oxidative stress was shown to promote liver fibrosis, cirrhosis, and cancer, as well as metabolic dysfunction. HIV-induced oxidative stress was shown to contribute to neurodegenerative complications which are often observed in AIDS patients. Last but not least, a virus-induced oxidative burst has been recently associated with the development of the acute childhood lymphoblastic leukemia. In bacterial infections oxidative stress arises, at least in part, from altered metabolic pathways and has also been implicated in organ damage and the development of malignancies.* Helicobacter pylori*, for example, induces ROS-generating enzymes such as spermine oxidase and upregulates proinflammatory and procancerogenic redox-regulated genes like cyclooxygenase 2. Despite the overwhelming evidence of the role of oxidative stress in acute and chronic infection and the associated diseases, the impact of the majority of infectious agents on the host redox systems is not sufficiently characterized, with published data plagued by the controversies.

The current special issue aims to bring together both research papers and review articles on the mechanisms by which viral and bacterial infections trigger ROS production and induce and modulate the antioxidant defense systems and papers on the role of reactive oxygen species in viral propagation and infection-associated pathologies. We have also invited manuscripts from other fields of redox biology in order to bring new perspectives to the studies of oxidative stress in infections and consequent disease.

Contributed manuscripts emphasize the heavy impact of oxidative stress on the pathogenesis of viral infections. Two independent reviews discuss oxidative stress in chronic infection with hepatitis C virus (HCV). The review by R. Medvedev et al. scrutinizes multiple mechanisms by which HCV enhances ROS production providing at the same time an in-depth analysis of viral influence on the protective Nrf2 pathway. Special attention is paid to the redox-regulated events by which HCV triggers autophagy such as ER stress and the unfolded protein response. A concise review article of K. Rebbani and K. Tsukiyama-Kohara focuses on ROS-sensitive mechanisms of HCV-induced hepatocarcinogenesis. In particular, this review discusses the cholesterol biosynthesis enzyme 3*β*-hydroxysterol Δ24-reductase (DHCR24), induced by HCV and recognized as a host marker of oxidative stress, as biomarker for the prognosis of hepatocellular carcinoma. These reviews are complemented by a research paper by O. A. Smirnova et al. on the mechanisms of induction of oxidative stress by a component of HCV replication complex nonstructural protein NS5A. Earlier NS5A was thought to induce oxidative stress by altering calcium homeostasis [1]. In contrast to this, O. A. Smirnova et al. found that NS5A contributes to ROS production by activating expression of NADPH oxidases 1 and 4 as well as cytochrome P450 2E1. NOX1 and NOX4 induction was mediated by enhanced production of transforming growth factor *β*1. Earlier similar results were obtained by us for HCV core protein [2]. This perfectly exemplified the capacity of the virus to induce oxidative stress by several multistep mechanisms.

One review and an experimental manuscript deal with oxidative stress in HIV infection. A review by the editors of this issue summarizes the data on the oxidative stress markers associated in HIV infection, analyzes mechanisms by which this virus triggers massive ROS production, and scrutinizes the scarce data that exist on the effect of ROS on HIV-1 replication. The review presents the current state of knowledge on the redox alterations as crucial factors of HIV-1 pathogenicity, namely, neurotoxicity, dementia, and T-cell exhaustion, as well as certain side effects of the antiretroviral therapy, in comparison to the pathologies associated with the nitrosative stress. A thorough experimental work by X. Huang et al. analyzes the status of oxidative stress in HIV/HCV coinfection delineating the contribution of HIV to HCV-induced liver damage. The study also discusses correlations between liver pathology and level of oxidative stress in HIV/HCV-coinfected patients, as well as the capacity of HIV infection to accelerate HCV-associated liver damage in HIV/HCV-coinfected individuals.

Not only RNA, but also a variety of DNA viruses are associated with the increased oxidative stress promoting DNA damage, high mutagenicity, and initiation and/or progression of neoplasia [3]. A review article by M. Mushtaq et al. summarizes current knowledge on the metabolic disturbances induced by DNA viruses, many of which are achieved through the redox-sensitive processes. This review focuses on the enhanced consumption of glucose, its conversion into pyruvate (glycolysis), and the switch to lactate formation, characteristic for cells transformed with tumor DNA viruses. Under physiological conditions, one glucose molecule is converted into two pyruvate molecules. Oxidation of pyruvate to CO_2_ and O_2_ results in synthesis of 38 ATP molecules. At low oxygen concentrations, pyruvate is not oxidized but instead converted to lactate; this process still produces ATP but is independent of O_2_ and can occur under hypoxic conditions. Glucose is not only important for ATP production, but also used by the pentose phosphate pathway to produce nucleic acids and NADPH which in turn required to neutralize ROS. Cancer cells increase frequently glucose uptake and lactate production, which are features of the Warburg effect [4, 5]. The review by M. Mushtaq et al. summarizes evidence that shows that not only tumor DNA viruses, but also single viral proteins can enhance glucose uptake and control the tumor microenvironment, thus promoting tumor growth and metastasis.

While six papers of the special issue deal with the oxidative stress and consequent disease caused by viral infections, G. S. Krasnov et al. uncover the changes in transcriptome in the tumor tissues from patients with colorectal cancer associated with infection with* Bacteroides fragilis*. This study reveals a significant induction of a number of enzymes involved in the biosynthesis and degradation of biogenic polyamines including spermine oxidase (SMO). SMO converts spermine into spermidine with formation of H_2_O_2_ and acrolein as by-products [6]. Earlier studies associated this enzyme with the development of gastrointestinal tumors in patients infected with both* Bacteroides fragilis* [7] and* Helicobacter pylori* [8]. We believe that such a modulation of the polyamine metabolism is also highly relevant to viral infections. Indeed, we have recently shown similar induction of SMO by hepatitis C virus and its proteins [9]. However, the impact of changes to the polyamine metabolism on the physiopathology associated with viral infections remains to be studied.

Autoimmune arthritis can be induced experimentally; however, in human disease, both viral and bacterial infectious agents have been extensively discussed as triggers [10–12]. In this special issue, one paper deals with autoimmune arthritis, albeit induced by an experimental immunization. A. A. Andreev-Andrievskiy et al. present the evidence of promising therapeutic activity of the mitochondrially targeted antioxidant SkQ against rheumatoid arthritis experimentally induced by immunizing rats with collagen. In our view, a treatment approach based on the usage of antioxidants could be applied to prevent infection-associated pathologies and deserves experimental testing in the relevant animal models of human infections.

Finally, a very interesting paper of V. A. Mitkevich et al. discusses glutathionylation of Na,K-ATPase. The paper shows that this redox-sensitive posttranslational modification is promoted by hypoxia, an event accompanied by a severe oxidative stress. The authors provide evidence that the glutathione residues are shielding by the protein inside the protein globule. This paper, even though it does not deal with an infectious agent, may be mechanistically highly applicable to future studies in redox biology of human pathogens. To our knowledge, there is almost no published data on the redox-sensitive modifications of bacterial or viral proteins, with the exception of HIV protease.

We hope that this issue will help to deepen our understanding of the role of redox homeostasis in the pathologies associated with human infection by drawing attention to the novel aspects of redox control, such as the polyamine metabolism, glutathionylation, and novel therapies based on the use of antioxidants.
